# Comparison of Framingham 10-year cardiovascular risks in Sweden- and foreign-born primary health care patients in Sweden

**DOI:** 10.1186/s12889-023-15449-6

**Published:** 2023-03-22

**Authors:** Marina Taloyan, Victor Wågström, Kristin Hjörleifdottir Steiner, Danial Yarbakht, Claes-Göran Östenson, Helena Salminen

**Affiliations:** 1 Division of Family Medicine and Primary Care, Department of Neurobiology, Care Sciences and Society (NVS), Karolinska Institutet, Alfred Nobels allé 23, Huddinge, S-141 83 Stockholm, Sweden; 2 Academic Primary Health Care Centre, Stockholm County Council, Box 45436, 104 31 Stockholm, Sweden; 3 Region Stockholm, Karolinska Hospital, Solna, Sweden; 4 Department of Molecular Medicine and Surgery, Endocrine and Diabetes Unit, Karolinska Institutet, Solna, Sweden

**Keywords:** Cardiovascular risk factors, Framingham risk score, Primary care, Region of birth, Sweden

## Abstract

**Background:**

The prevalence of cardiovascular disease around the world varies by ethnicity and region of birth. Immigrants living in Sweden may have a higher prevalence of cardiovascular diseases than native-born Swedes, but little is known about their actual cardiovascular risk. This study aimed to examine the relationship in Sweden between 10-year cardiovascular risk and birthplace.

**Method:**

This cross-sectional study was based on cardiovascular risk factor data obtained from the 4D Diabetes Project, a Programme 4D subproject in Sweden. Participants were recruited from two primary healthcare centres in Stockholm without a history of diabetes or pre-diabetes. The outcome variable was 10-year cardiovascular risk based on the calculation of a Framingham Risk Score with six risk factors: age, sex, LDL, HDL, BP, diabetes and smoking for each participant. Multiple linear regression was performed to generate β-coefficients for the outcome.

**Results:**

There was an average of 8.86% cardiovascular risk over 10 years in Sweden-born participants and a 5.45% 10-year risk in foreign-born, (*P* < 0.0001). Foreign-born participants were about 10 years younger (mean age 46 years vs. 56 years, *P* < 0.001), with a significantly higher proportion of smokers (23.9% vs. 13.7%; *P* = 0.001). To be born in Sweden (with parents born in Sweden) was significantly associated with a 10-year cardiovascular risk in the crude model (β- coefficient = 3.40, 95% CI 2.59–4.22; *P* < 0.0001) and when adjusted for education and alcohol consumption (β- coefficient = 2.70 95% CI 1.86–3.54; *P* < 0.0001). Regardless of the birthplace, 10-year cardiovascular risk was lower for those with higher education compared to those with less than 10 years of education.

**Conclusion:**

This study found a relationship between 10-year calculated cardiovascular risk and place of birth. Sweden-born participants had a higher association with 10-year cardiovascular risk than foreign-born participants. These results contradict previous reports of higher rates of CVD in residents of Middle-Eastern countries and Middle-Eastern immigrants living in Sweden.

## Introduction

Cardiovascular disease (CVD) is the leading cause of death globally, responsible for an estimated 17.9 million deaths annually, and contributing to one-third of all deaths in the world [1, 2]. An important non-modifiable risk factor for CVD is a genetic preposition, which includes sex [3]. Nevertheless, modifiable risk factors also play a huge role in CVD, as demonstrated by the fact that they are estimated to collectively cause more than half of all cardiovascular deaths [4]. In a study of patients from 52 countries, more than 90% of all first-time myocardial infarctions were attributable to one or more of nine modifiable risk factors, including tobacco smoking, hypertension, dyslipidaemia, diabetes, obesity, psychosocial factors, inadequate physical activity, alcohol consumption, and poor diet [3, 5].

Various combinations of risk factors have been used to calculate CVD risk scores, including the Systematic Coronary Risk Evaluation (SCORE2) which is recommended for use in the Swedish population, Prospective Cardiovascular Münster (PROCAM), Turkish Adult Risk Factor Study (TEKHARF), and Framingham (FRS) risk scores. The scores are then used to estimate the risk of a patient having cardiovascular events (SVE) within a designated period. The FRS combines several well-established cardiovascular risk factors to determine an estimation of the risk of having a CVE within 10 years [6–8]. It can be used to guide whether to initiate suitable risk-reducing therapies, such as a statin or anti-hypertensive medications, as part of clinical care. A particular strength of the FRS is that it considers both systolic and diastolic blood pressure (BP), as well as both LDL-C and high-density lipoprotein (HDL) levels [8], whereas, for example, SCORE2 considers only systolic BP and non-HDL cholesterol levels [9].

The prevalence of CVD, around the world, measured in a variety of ways, varies by ethnic background and region of birth [10–12]. Within Sweden, the prevalence of CVD in different immigrant populations also varies substantially [13–15], but most of the studies reporting those results are now more than a decade old and showed contradictory higher or lower CVD mortality. One of those observed lower mortality risk of Swedish residents born in countries with high CVD mortality (for ex. Finland and Hungary) and a higher risk for immigrants born in low-risk CVD countries in South Europe than in their countries of birth [16]. However, another study noted that immigrants living in Sweden had a lower prevalence of CVD than comparable populations in their countries of birth [17]. In contrast, a 2016 publication reported that non-western immigrants with type 2 diabetes living in Sweden had a significantly lower risk of CVD-related mortality than native Swedes [13]. These studies suggest that immigrant populations living in Sweden may have a higher prevalence of CVD, but a lower risk of CVD-related death, than the native-born Swedish population. With these contradictory observations in mind, there appears to be a need for an updated evaluation of CVD and 10-year cardiovascular risk estimates in immigrant populations living in Sweden.

The primary aim of this study was to examine the relationship between having a 10-year cardiovascular risk and birthplace (Sweden or foreign), in primary healthcare patients living in Sweden. The secondary aim was to determine whether there were any significant associations in these populations between 10-year cardiovascular risk and other potential CVD risk factors that are not part of the FRS, such as level of education, physical activity, alcohol consumption, and hs-CRP levels.

## Methods

### Study population

This cross-sectional study was based on data obtained from the ‘4D Diabetes Project: Screening and Treatment of Prediabetes and Diabetes in Primary Care – A Pilot Study’, a Programme 4D sub-project conducted by the Karolinska Institutet and Region Stockholm (former Stockholm County Council). Participants were recruited for the study from two primary healthcare centres (PHCC) in Stockholm. Participants were offered inclusion in the study if they were between 18 and 74 years old and had no previous history of diabetes or pre-diabetes. The Jakobsberg and Flemingsberg PHCCs were selected because large portions of their patient populations were born outside of Sweden, particularly outside of Europe.

### Data acquisition

Data were collected from participants at the two PHCCs from 2013 through 2015. At the first visit, each participant was interviewed to obtain information about demography, country of birth, level of education, and self-reported health and lifestyle habits (physical exercise, smoking, and alcohol consumption). During that visit, BP was measured, and a capillary haemoglobin A1c (HbA1c) test was performed. When participants returned for a second visit, fasting blood samples were obtained, and these were analysed for serum levels of total cholesterol, HDL, calculated LDL-C, triglycerides, and hs-CRP. Also, another BP measurement was taken. For our study, the mean values of the two BP readings were used. Each participant with an HbA1c ≥ 39 mmol/mol at the first visit was offered an oral glucose tolerance test (OGTT).

### Participant characteristics

For participant characteristics that represented non-Framingham CVD risk factors, we converted some of the raw data into categorical variables. Level of education categories were 0 to 9 years, 10 to 12 years, and more than 12 years. Physical exercise initially had five response alternatives, which we collapsed into three categories: no physical activity at all, more than 0 min and up to 60 min per week, and more than 60 min per week. Alcohol consumption categories were less than 4 glasses a week and 4 glasses or more per week. Smoking initially had seven response options, which we collapsed into two categories: quit or never smoked and any active smoking. Diabetes had two categories: yes and no.

### Framingham risk factors

We calculated a Framingham Risk Score (FRS) for each participant, based on six risk factors, including age, sex, LDL-C, HDL, BP, diabetes, and smoking. According to FRC calculation total points for females and males were based on different points: for females: from -2 to 14 and for males: from -3 to 14. Using the system described by Viera and Sheridan, points were either awarded or subtracted for each risk factor based on being above or below cut-offs, which included the following: (a) age 35–39 years in men, 40–44 years in women; (b) LDL-C of 2.59 mmol/L to 4.13 mmol/L for both men and women; (c) HDL of 1.16 mmol/L to 1.54 mmol/L in men and 1.29 mmol/L to 1.54 mmol/L in women; (d) BP of 120/80 to 129/84 in men and 120/80 to 139/89 in women; (e) presence of diabetes; and f) smoking [7]. A 10-year cardiovascular risk was then determined using total points. We did a calculation of the total points of FRC for the entire study population and by sex separately (Table 3).

### Outcome characteristics

For this study, we used the 10-year cardiovascular risk based on FRC in a regression analysis as a continuous variable.

### Birthplace groups

As an independent variable, we divided the participants in this study into two groups based on birthplace: (1) Sweden-born, comprised of those who were born in Sweden, with both parents also born in Sweden; and (2) Foreign-born, comprised of those who were born outside of Sweden or had at least one parent born outside of Sweden. This was based on the official definition of foreign-born used in Sweden [18].

### Statistical methods

Descriptive statistics involving the prevalence of demographic, clinical, and outcome characteristics are presented as frequencies and proportions, and those involving Framingham risk factor characteristics are presented as means and standard deviations (SD). Comparisons were performed using Chi-square tests for categorical variables, T-tests for continuous variables following a normal distribution, and Wilcoxon-Rank sum tests for continuous variables not following a normal distribution. For continuous variables, 95% Confidence intervals (CI) were calculated when means were compared. Multiple linear regression analyses were performed to generate unadjusted β-coefficients and 95% CI for the outcome of 10-year cardiovascular risk and to generate adjusted β-coefficients for education and alcohol consumption. Additionally, were β-coefficients and 95% CI generated stratified separately for age groups, males and females and are presented in three tables. A *P* value of 0.05 or less was considered significant. Calculations were performed using STATA statistical software version 17.0 [19].

## Results

A total of 830 participants were included in the study. These participants were born in Sweden with Swedish-born parents (*n* = 278) and outside Sweden (*n* = 552) with the largest portion born in the Middle East, including 86 in Turkey, 52 in Iran, and 48 in Iraq.

### Participant and outcome characteristics

Table 1 presents descriptive statistics for participant characteristics and outcome measures. Foreign-born participants had significantly lower 10-year cardiovascular risk compared to Sweden-born participants (mean 5.45 vs. 8.86 respectively (*P* < 0.0001). Additional significant differences were found between the studied groups in age, education, and alcohol consumption.

**Table 1:** Participant and outcome characteristics of 830 participants, by birthplace, Stockholm, Sweden, 2013–2015

*Characteristics*	Sweden-born n (%)	Foreign-born n (%)
Participants	278 (33.5)	552 (66.5)
Age,^*^ *years*		
≤ 40	47 (16.9)	206 (37.3)
41–50	41 (14.8)	130 (23.6)
51–60	56 (20.1)	138 (25.0)
> 60	134 (48.2)	78 (14.1)
Sex		
Male	122 (43.9)	233 (42.2)
Female	156 (56.1)	319 (57.8)
Education^*^,* years*		
< 9	43 (15.5)	105 (19.3)
> 9 to 12	102 (36.7)	128 (23.5)
> 12	133 (47.8)	312 (57.3)
Physical Activity, *minutes per week*		
None	147 (53.1)	295(53.9)
0 to 60	56 (20.2)	126 (23.0)
> 60	74 (26.7)	126 (23.1)
hs-CRP^a^, *mmol/L*		
≤ 3	232 (83.5)	466 (84.4)
> 3	46 (16.5)	86 (15.6)
Alcohol consumption, glasses per week^*^		
< 4	199 (71.6)	511 (95.6)
≥ 4	79 (28.4)	41 (7.4)
Framingham 10-year elevated cardiovascular risk^*^		
Mean, SD 95%	8.86 (8.00—9.72)	5.45 (5.07—5.84)
Framingham 10-year elevated cardiovascular risk (cut off 10), n ( %)		
≥ 10	99 (35.6)	71 (12.9)
< 10	179 (64.4)	481 (87.1)

^*^P < 0.0001 (chi-square or T-test)

^a^high-sensitivity C-reactive protein

### Framingham risk characteristics

In total, there were statistically significant differences in all variables included in Framingham risk score between the two studied groups. Compared to Sweden-born participants, estimated mean or median values for foreign-born participants were significantly lower for age, LDL, HDL and systolic and diastolic blood pressure levels (Table 2). Conversely, a significantly higher percentage of foreign-born participants were smokers.

**Table 2:** Framingham risk factor characteristics for all 830 participants and by birthplace, Stockholm, Sweden, 2013–2015

***Risk factor characteristics***^a^	**Total (*N* = 830)**	**Sweden-born (*n* = 278)**	**Foreign-born (*n* = 552)**
Age, *years,mean (SD)*	49.0 ± 14.6	55.8 ± 15.0	45.6 ± 13.3^*^
LDL-C, *mg/dL, mean (SD)*	3.0 ± 0.9	3.1 ± 0.9	3.0 ± 0.9^***^
HDL, *mg/dL, mean (SD)*	1.4 ± 0.4	1.5 ± 0.4	1.3 ± 0.4^*^
Systolic BP, *mm Hg mean (SD)*	121.4 ± 17.4	128.4 ± 18.3	117.8 ± 15.8^*^
Diastolic BP, *mm Hg mean (SD)*	76.6 ± 10.2	78.2 ± 10.4	75.8 ± 10.0^**^
Smoking^b^, *n (%)*	170 (20.5)	38 (13.7)	132 (23.9)^**^

*Abbreviations*: *LDL-C* Low-density lipoprotein cholesterol, *HDL* High-density lipoprotein, *BP* Blood pressure, *SD* Standard deviation

^*^P < 0.0001

^**^P < 0.001

^***^P < 0.05

^a^Diabetes not listed because no participants had diabetes

^b^Chi2-test

### Framingham risk characteristics by sex and place of birth

Significant differences in total Framingham risk score components between females and males in the entire study population were observed (Table 3). Females had higher mean levels of HDL and lower mean levels of both diastolic and systolic BP than males.

**Table 3:** Framingham risk factor characteristics for study population by sex, Stockholm, Sweden, 2013–2015

***Risk factor characteristics***^a^	**Total (*N* = 830)**	**Female (*n* = 475)**	**Male (*n* = 355)**
Age, *years mean (SD)*	49.0 ± 14.6	48.6 ± 14.9	49.5 ± 14.2
LDL-C, *mg/dL mean (SD)*	3.0 ± 0.9	3.0 ± 0.8	3.1 ± 0.9
HDL, *mg/dL mean (SD)*	1.4 ± 0.4	1.5 ± 0.4	1.2 ± 0.3^*^
Systolic BP, *mm Hg mean (SD)*	121.4 ± 17.4	119.4 ± 18.3	124.0 ± 15.7^**^
Diastolic BP, *mm Hg mean (SD)*	76.6 ± 10.2	74.9 ± 9.7	78.8 ± 10.5^*^
Smoking*, n (%)*	170 (20.5)	89 (18.7)	81 (22.8)
Total Framingham risk, mean, SD 95%	6.60 (6.20—6.96)	4.95 (4.59—5.31)	8.80 (8.06—9.55)^*^

*Abbreviations*: *LDL-C* Low-density lipoprotein cholesterol, *HDL* High-density lipoprotein, *BP* Blood pressure, *SD* Standard deviation

^*^P < .0001

^*^^*^P < .001

^a^Diabetes not listed because no participants had diabetes

In the Sweden-born population, females had significantly higher mean levels of HDL and lower mean levels of diastolic BP than males (Table 4). In the foreign-born population, females had significantly higher mean levels of HDL and they had lower mean levels of systolic and diastolic BP than males (Table 5). In this population, a significantly lower percentage of females were smokers.

**Table 4:** Framingham risk factor characteristics for 278 Sweden-born participants, by sex, Stockholm, Sweden, 2013–2015

***Risk factor characteristics***^a^	**Total Sweden-born (*N* = 278)**	**Female (*n* = 156)**	**Male (*n* = 122)**
Age, *years mean (SD)*	55.8 ± 15.0	54.9 ± 15.5	56.8 ± 14.3
LDL-C, *mg/dL mean (SD)*	3.1 ± 0.9	3.2 ± 0.9	3.1 ± 0.9
HDL, *mg/dL mean (SD)*	1.5 ± 0.4	1.6 ± 0.4	1.3 ± 0.3^*^
Systolic BP, *mm Hg mean (SD)*	128.4 ± 18.3	126.6 ± 19.4	130.8 ± 16.5
Diastolic BP, *mm Hg*	78.2 ± 10.4	76.4 ± 10.1	80.6 ± 10.4^**^
Smoking, *n (%)*	38 (13.7)	26 (16.7)	12 (9.8)

*Abbreviations*: *LDL-C* Low-density lipoprotein cholesterol, *HDL* High-density lipoprotein, *BP* Blood pressure, *SD* Standard deviation

^*^P < .0001

^**^P < .001

^a^Diabetes not listed because no participants had diabetes

**Table 5:** Framingham risk factor characteristics for 552 foreign-born participants, by sex, Stockholm, Sweden, 2013–2015

*Risk factor characteristics*^*a*^	*Total* *Foreign-born* (*N* = 552)	*Female* (*n* = 319)	*Male* (*n* = 233)
*Age, years mean (SD)*	45.6 ± 13.3	45.5 ± 13.7	45.7 ± 12.7
*LDL-C, mg/dL mean (SD)*	3.0 ± 0.9	3.0 ± 0.8	3.0 ± 0.9
*HDL, mg/dL mean (SD)*	1.3 ± 0.4	1.5 ± 0.4	1.2 ± 0.3^*^
*Systolic BP, mm Hg mean (SD)*	117.8 ± 15.8	115.8 ± 16.7	120.6 ± 14.1^**^
*Diastolic BP, mm Hg mean (SD)*	75.8 ± 10.0	74.3 ± 9.5	77.9 ± 10.4^*^
*Smoking, n (%)*	132 (23.9)	63 (19.8)	69 (29.6)^***^

*Abbreviations*: *LDL-C* Low-density lipoprotein cholesterol, *HDL* High-density lipoprotein, *BP* Blood pressure, *SD* Standard deviation

^*^P < .0001

^**^P < .0004

^***^P < .007

^a^Diabetes not listed because no participants had diabetes

### Risk factors for 10-year cardiovascular risk

Results of regression analyses showed that Sweden-born participants had a significant association of risk of 10-year cardiovascular risk in a crude model (β-coefficient = 3.40 95% CI 2.59–4.22; *P* < 0.0001) and when adjusted for education and alcohol consumption (β- coefficient = 2.70 95% CI 1.86–3.54; *P* < 0.0001) compared to foreign-born participants (Table 6). Regardless of the birthplace, 10-year cardiovascular risk was lower for those with higher education than for those with less than 10 years of education. Finally, those who consumed more than 4 glasses of alcohol had a higher association than those with less alcohol consumption (β-coefficient = 3.27, 95% CI 2.14–4.40; *P* < 0.0001).

**Table 6:** β- coefficients^a^  for 10-year cardiovascular risk with cardiovascular risk with 95% Confidence Intervals (CI), Stockholm, Sweden, 2013–2015

**Variables**	**Unadjusted**^b^	** + education**	** + alcohol**
Place of birth			
Foreign-born	0.00 (Ref)	0.00 (Ref)	0.00 (Ref)
Sweden-born	3.40 (2.59—4.22)^*^	3.42 (2.61—4.24)^*^	2.70 (1.86—3.54)^*^
Education, years			
< 9		0.00 (Ref)	0.00 (Ref)
9 to12		-2.04 (-3.20—.88)^**^	-2.00 (-3.12—.85)^**^
> 12		-2.41 (-3.45—-1.37)^*^	-2.44 (-3.46—-1.42)^*^
Alcohol consumption, glasses a week			
≤ 4			0.00 (Ref)
> 4			3.27 (2.14—4.40)^*^

^*^P < .0001

^*^^*^P < .001

^a^Linear regression analysis done using stepwise adjusting for independent variables, only covariates with statistically significant prevalence (see Table 1) and coefficients included in this Table

^b^Unadjusted (crude) β-coefficients; all other results in column are adjusted coefficients

Tables 7 and 8 present β-coefficients for 10-year cardiovascular risks, by place of birth with foreign-born as reference stratified for males and females separately. Significant associations were observed in the Sweden-born group when adjusted for education and alcohol in both males (β-coefficient = 4.18, 95% CI 2.64–5.72; *P* < 0.0001) and females (β-coefficient 1.92, 95% CI 1.16–2.70; *P* < 0.0001) compared to foreign-born participants.

**Table 7:** β-coefficients^a^ for 10-year cardiovascular risk with 95% Confidence Intervals (CI), by place of birth with foreign-born as reference for males only, *n* = 355

Variables	Unadjusted^b^	+ education	+ education, alcohol
Place of birth			
Foreign-born	0.00 (Ref)	0.00 (Ref)	0.00 (Ref)
Sweden-born	4.98 (3.50—6.46)^*^	4.93 (3.45—6.40)^*^	4.18 (2.64—5.72)^*^
Education, years			
< 9		0.00 (Ref)	0.00 (Ref)
9 to12		**-**3.43 (-5.65—-1.20)^**^	**-**3.33 (-5.54—-1.13)^**^
> 12		**-**3.69 (-5.69—-1.70)^*^	**-**3.64 (-5.62—-1.67)^*^
Alcohol consumption, glasses a week			
≤ 4			0.00 (Ref)
> 4			2.63 (0.87—4.39)^**^

^*^P < .0001

^**^P < .001

^a^Linear regression analysis done using stepwise adjusting for independent variables, only covariates with statistically significant prevalence (see Table 1) and coefficients included in this Table

^b^Unadjusted (crude) β-coefficients; all other results in column are adjusted coefficients

**Table 8:** β-coefficients^a^ for 10-year cardiovascular risk with 95% Confidence Intervals (CI), by place of birth with foreign-born as reference for females only, *n* = 475

Variables	Unadjusted^b^	+ education	+ education, alcohol
Place of birth			
Foreign-born	0.00 (Ref)	0.00 (Ref)	0.00 (Ref)
Sweden-born	2.08 (1.34—2.82)^*^	2.09 (1.35—2.8^3)*^	1.92 (1.16—2.70)^*^
Education, years			
< 9		0.00 (Ref)	0.00 (Ref)
9 to12		-1.34 (-2.34—.32)^**^	-1.33 (-2.34—.32)^**^
> 12		-2.11 (-3.01—-1.20)^*^	-2.12 (-3.03—-1.22)^*^
Alcohol consumption, glasses a week			
≤ 4			0.00 (Ref)
> 4			0.97 (-.33—-2.29)

^*^P < .0001

^**^P < .05

^a^Linear regression analysis done using stepwise adjusting for independent variables, only covariates with statistically significant prevalence (see Table 1) and coefficients included in this Table

^b^Unadjusted (crude) β-coefficients; all other results in column are adjusted coefficients

Stratification for age groups generated β-coefficients for 10-year cardiovascular risks, by place of birth with foreign-born as reference is shown in Table 9. Regardless of the birthplace, significantly lower associations were observed in the group aged 41–50 with the highest education compared to lower education (β-coefficient = -1.38 95% CI -2.65-0.12; *P* < 0.05) and association with higher estimated 10-year cardiovascular risk in the group aged 51–60 who consumed at least 4 glasses of alcohol a week compared to those who consumed fewer than 4 glasses weekly (β-coefficient = 3.30 95% CI 1.18–5.40; *P* < 0.05). There were no significant differences between Swedish- and foreign-born participants in cardiovascular risk in age-stratified analyses.

**Table 9:** β-coefficients for 10-year cardiovascular risk with 95% Confidence Intervals (CI), by place of birth with foreign-born as reference stratified for age groups

Variables	Age 18–40 *N* = 253	Age 41–50 *N* = 171	Age 51–60 *N* = 194	Age > 61 *N* = 212
Place of birth				
Foreign-born	0.00 (Ref)	0.00 (Ref)	0.00 (Ref)	0.00 (Ref)
Sweden-born	-.21 (-.64—.21)	0.92 (-.22—2.06)	.49 (-1.21—2.20)	1.81 (-.30—3.93)
Education, years				
< 9	0.00 (Ref)	0.00 (Ref)	0.00 (Ref)	0.00 (Ref)
9 to12	.01 (-.66—.68)	-1.12 (-2.63—.40)	.27 (-1.83—2.36)	-1.56 (-4.16—1.04)
> 12	.10 (-.53—.73)	-1.38 (-2.65—-.12)^*^	-.46 (-2.33—1.42)	-.73 (-3.16—1.71)
Alcohol consumption, glasses a week				
≤ 4	0.00 (Ref)	0.00 (Ref)	0.00 (Ref)	0.00 (Ref)
> 4	.10 (-.74—.77)	0.67 (-1.13—2.47)	3.30 (1.18—5.40)^*^	1.52 (-.73—3.78)

^*^P < .05

The results of regression in four groups by birthplace showed a similar pattern: those born in Iraq, Iran or Turkey had a significantly lower association of a 10-year cardiovascular risk than Sweden-born (Table 10).

**Table 10:** β-coefficients^a^ for 10-year cardiovascular risk with 95% Confidence Intervals (CI), by place of birth in Iraq, Iran, and Turkey with Sweden-born as reference

Variables	Unadjusted	+ education, alcohol
Place of birth		
Sweden-born	0.00 (Ref)	0.00 (Ref)
Born in Iraq	- 4.22 (-6.01—-2.43)^*^	-3.00 (-4.85—-1.15)^**^
Born in Iran	-3.24 (-5.08—-1.41)^**^	-1.92 (-3.82—-.007)^***^
Born in Turkey	-3.38 (-4.90—-1.88)^**^	-3.67 (-5.41—-2.10)^*^
Education, years		
< 9		0.00 (Ref)
9 to12		-2.70 (-4.40—-.99)^***^
> 12		-3.10 (-4.72—-1.48)^*^
Alcohol consumption, glasses a week		
≤ 4		0.00 (Ref)
> 4		3.02 (1.48—4.56)^*^

^*^P < .0001

^**^P < .001

^***^P < .05

^a^Linear regression with unadjusted (crude) β-coefficients and all adjusted coefficients (in column + education and alcohol)

## Discussion

In this cross-sectional study, we examined the relationship among patients in Swedish PHC without a diagnosis of diabetes between 10-year cardiovascular risk and birthplace. The main finding was that after controlling for other variables, participants born in Sweden with parents born in Sweden had an association with higher 10-year cardiovascular risk compared with foreign-born participants. In addition, lower levels of education and higher alcohol consumption were significantly associated with the 10-year cardiovascular risk, regardless of birthplace.

The finding in our study was inconsistent with other published studies, which have reported higher rates of CVD in the Middle-East countries than in countries in Western Europe, including Sweden [10–15, 20]. In our study, foreign-born participants had significantly lower systolic and diastolic BP levels than Sweden-born participants, which is similar to findings in other studies [14, 15]. Foreign-born participants also had significantly lower LDL-C levels, and they were on average 10 years younger than the Sweden-born participants.

In general, it has been shown in a study performed in 52 countries that there are nine modifiable risk factors for first-time myocardial infarction including psychosocial factors and alcohol consumption [3, 5]. However, there are biological factors that have an impact on cardiovascular risks such as age. The fact that blood lipids (LDL) and blood pressure are highly age-dependent may explain the significant differences in average percentages of risk of 10 years cardiovascular risks between the studied groups. Performed stratified age analyses showed only two significant results: regardless of country of birth, higher education and less alcohol consumption a week were associated with lower 10-year cardiovascular risk in the groups aged 41–50 and 51—60 respectively. There were no significant differences between Swedish- and foreign-born participants in cardiovascular risk in age-stratified analyses. Thus, we can conclude that differences in age between the groups in cardiovascular risk can explain the differences in cardiovascular risks to a large extent.

Previous studies in Sweden on cardiovascular health in foreign-born citizens have largely focused on single countries of birth, such as Iraq [14, 15, 20]. We thought that the inclusion of participants with a wider variety of places of birth might make our results more applicable to immigrant populations throughout Sweden. However, we also acknowledge that differences in the cardiovascular health and risk profiles of individual foreign-born participants, even between those from different countries within each region, may limit the generalizability of our results to all foreign-born people in Sweden.

The issue of cardiovascular risk in immigrant populations in Sweden has received very little attention, despite several studies suggesting that the prevalence of CVD in these populations may be substantial [10–15, 20]. Studies comparing Middle Eastern and Western countries (including Sweden) have consistently found a higher prevalence of CVD in the Middle East [16, 17]. Other studies have reported a higher prevalence of CVD for immigrants with or without diabetes living in Sweden who are from the Middle East [15, 19, 20], though a lower prevalence of CVD when those immigrants are compared to populations in their native countries [21]. However, a more recent report of Swedish residents suggested that CVD-related mortality risk was lower for non-Western-born immigrants than native Swedes [18]. Our study showed that participants born in Sweden were more likely to have a higher average of 10-year cardiovascular risk than those who were foreign-born. Although these studies all looked at subtly different outcomes for example Bennet and colleagues explored CVD events and CVD mortality and thus not answering the same questions, these outcomes were all closely linked to cardiovascular health. As such, the contradictory results of these studies, including ours, call into question whether the cardiovascular risk scoring systems used for native populations in Western countries apply to immigrant populations from the Middle East.

The initial Framingham Heart Study from 1948 looked at a predominantly white population of European descent but subsequent studies in 1994 and 2003 enrolled more ethnically diverse individuals [21]. The Framingham criteria used for estimating 10-year cardiovascular risk in our study was based on this more diverse data, but they still lack any adjustments for different countries, regions of birth, or ethnicities [7, 8]. The contradiction between our study results and the known high prevalence of CVD in Middle Eastern populations suggests that there may be considerable value in conducting larger, longitudinal, and multigenerational studies, similar to the Framingham Heart Study, either in the Middle East or of Middle-Eastern immigrants in Sweden, or both, to construct new cardiovascular risk assessment tools or thresholds for this population.

It is worth emphasizing that our findings are not representative of the entire Swedish population of primary healthcare patients since data were collected only at two PHCCs. However, it would be informative to conduct multi-generational studies of immigrant populations to determine whether cardiovascular risk and prevalence become more like that of native-born populations over time. The healthy migrant effect might influence health advantages in immigrants, however, in our sample, we lack information on the duration of residence in Sweden and the participants’ health status in their countries of origin before they emigrated to Sweden [22]. Differences between the studied groups might additionally be explained by the fact that there were healthier immigrants and less healthy Sweden-born participants living in the selected settlements (Flemingsberg and Jakobsberg) than in the other areas of Stockholm or Sweden.

Regardless, our results suggest that a unique cardiovascular risk scoring system modified for the region of birth or ethnicity may be needed in Sweden. It may be prudent to update for healthcare providers to either look more closely at individual risk factors or apply a different threshold when making decisions about initiating CVD prevention or treatment interventions in patients with an immigrant background in Sweden. For example, a closer look at the Framingham risk factor results in our study suggests that smoking cessation programs and education about ways to increase HDL could potentially benefit those not born in Sweden. Taking this type of approach may help improve awareness about CVD and expand access to preventative interventions for immigrant populations in Sweden.

### Limitations

The data used in our study was dependent on the population specifically recruited for the 4D Diabetes Project in Sweden. This population involved two cluster samples, comprising all willing participants from two similar primary healthcare locations. Ultimately, the Sweden-born and foreign-born study population characteristics differed significantly, particularly in the mean ages of the participants. Given that age is particularly impactful on cardiovascular risk, we do not adjust for age in either linear or logistic regression analyses as age was included in the score. In addition, no drop-out analysis was conducted of those who chose not to accept recruitment into the project. Without information about the characteristics of those who chose not to participate, the possibility of other selection biases cannot be excluded. It is possible, for example, that Sweden-born and foreign-born patients had different reasons and thresholds for when they sought care at a PHCC. On a related note, another potential limitation of this study was that the data used was gathered from patients who were actively seeking care at a PHCC. These patients were more likely than the general public to be sick and/or have other underlying medical conditions, and this could affect the generalizability of our results to a broader population. Lastly, as this was a cross-sectional study, we were unable to ascertain causality in any of the associations. Therefore, the results and conclusions of our study can at most be used to deepen the knowledge base and generate new hypotheses for future studies.

## Conclusions

In this study, there was a relationship between 10-year calculated cardiovascular risk and place of birth. Sweden-born participants had a significant association with an estimated 10-year cardiovascular risk compared to the foreign-born participants. These results contradict previous reports of higher rates of CVD in residents of Middle-Eastern countries and Middle-Eastern immigrants living in Sweden. Regardless of the birthplace, healthcare providers might need to look more closely at alcohol consumption in patients with cardiovascular risks.

## Data Availability

Sharing the data with other researchers was not included in written informed consent; therefore, neither data nor materials are publicly available. The first author (MT) is the contact person in case of making a request.

## Abbreviations

BP: Blood pressure
β-coefficients: Beta coefficients
CVD: Cardiovascular disease
CVE: Cardiovascular event
FRS: Framingham Risk Score
HbA1c: Haemoglobin A1c
HDL: High-density lipoprotein
Hs-CRP: High-sensitivity c-reactive protein
LDL-C: Low-density lipoprotein-cholesterol
OGTT: Oral glucose tolerance test
OR: Odds ratio
PHCC: Primary Health care Centre
SD: Standard deviation
4D: 4 Diagnoses
